# Preparation and Properties of Antibacterial Silk Fibroin Scaffolds

**DOI:** 10.3390/polym15234581

**Published:** 2023-11-30

**Authors:** Peng Pan, Cheng Hu, Ahui Liang, Xueping Liu, Mengqi Fang, Shanlong Yang, Yadong Zhang, Mingzhong Li

**Affiliations:** National Engineering Laboratory for Modern Silk, College of Textile and Clothing Engineering, Soochow University, Suzhou 215123, China; ppanpanpeng@stu.suda.edu.cn (P.P.); hc928928@163.com (C.H.); lah892778661@163.com (A.L.); 18862107248@163.com (X.L.); 20214215051@stu.suda.edu.cn (M.F.); yangsl886886@163.com (S.Y.); 20215215066@stu.suda.edu.cn (Y.Z.)

**Keywords:** silk fibroin, scaffold, antibacterial materials, wound dressing, cytocompatibility

## Abstract

The development of a wound dressing with both antibacterial and healing-guiding functions is a major concern in the treatment of open and infected wounds. In this study, poly(hexamethylene biguanide) hydrochloride (PHMB) was loaded into a 3D silk fibroin (SF) scaffold based on electrostatic interactions between PHMB and SF, and PHMB/SF hybrid scaffolds were prepared via freeze-drying. The effects of the PHMB/SF ratio on the antibacterial activity and cytocompatibility of the hybrid scaffold were investigated. The results of an agar disc diffusion test and a bacteriostasis rate examination showed that when the mass ratio of PHMB/SF was greater than 1/100, the scaffold exhibited obvious antibacterial activity against *E. coli* and *S. aureus*. L-929 cells were encapsulated in the PHMB/SF scaffolds and cultured in vitro. SEM, laser scanning confocal microscopy, and CCK-8 assay results demonstrated that hybrid scaffolds with a PHMB/SF ratio of less than 2/100 significantly promoted cell adhesion, spreading, and proliferation. In conclusion, a hybrid scaffold with a PHMB/SF ratio of approximately 2/100 not only effectively inhibited bacterial reproduction but also showed good cytocompatibility and is expected to be usable as a functional antibacterial dressing for wound repair.

## 1. Introduction

Bacterial infection is one of the main problems associated with impaired wound healing [1]. When bacteria invade, the wound site provides a favorable environment for pathogen growth in terms of moisture, temperature, and nutrients, and bacteria can delay wound healing through different mechanisms. Bacteria consume nutrients and oxygen from tissues and produce endotoxins [2]. Bacterial infestation can lead to dilatation and hypoxia of the wound site, vascular occlusion, and ultimately local tissue death at the wound site [3,4]. Therefore, covering a wound with an antibacterial dressing to inhibit the reproduction of bacteria at the wound site and promote skin tissue regeneration is essential for wound healing.

Silk fibroin (SF) is a natural protein derived from silkworm silk, which is a rich source and easy to purify. SF is mainly composed of non-polar amino acids and neutral amino acids, among which Gly, Ala, and Ser account for 41.70%, 31.84%, and 10.78%, respectively [5]. The total content of polar amino acids in SF is only approximately 4.75%, among which the negatively charged Asp and Glu account for approximately 1.78% and 1.93%, respectively, and the positively charged Lys, Arg, and His account for 0.34%, 0.46%, and 0.25%, respectively, so SF is negatively charged in a neutral environment. SF has excellent biocompatibility, desirable mechanical properties, and adjustable biodegradability [6,7]. Following a skin injury, wound dressings fabricated using SF are able to regulate the expression levels of proinflammatory cytokines (IL-α, IL-6) and anti-inflammatory cytokines (IL-10) and control the expression of waveform proteins, fibronectin, cell cycle protein D1, and vascular endothelial growth factor by stimulating the NF-κB signaling pathway, thus promoting wound healing [8,9,10]. NF-κB signaling regulates cell growth and adhesion, blocks reactive oxygen generation, and promotes the healing of skin wounds [11,12]. Although SF-based wound dressings have significant positive effects on wound repair and healing, they lack the ability to inhibit bacterial reproduction on open wounds or on wounds with bacteria present. Therefore, endowing SF-based wound dressings with effective antibacterial functions is the key to their widespread application in the clinical treatment of open and bacteria-containing wounds.

Loading antimicrobials such as nano silver particles, antibiotics, antimicrobial peptides, and cationic polymers into wound dressings is an effective way of improving their antibacterial activity and preventing wound infection, but large doses of antimicrobials usually cause obvious side effects such as cytotoxicity and systemic toxicity [13,14,15]. For example, loading antimicrobial peptides into a chitosan/fibroin scaffold can improve inhibition against *E. coli* and *S. aureus*, but the viability of cells inoculated in the scaffold decreases from 97% to 70% [16]. Reducing the dose of antimicrobials can reduce the cytotoxicity of wound dressings, but the antibacterial ability is also decreased [17]. The selection of antibacterial agents, the method of loading them into a wound dressing, and the way of binding them to the dressing have significant effects on the antibacterial activity and cytocompatibility of the dressing [18].

Poly(hexamethylene biguanide) hydrochloride (PHMB) is a cationic oligomer containing an average of 7–11 biguanide groups separated by flexible hexamethylene chain segments [19]. PHMB is an environmentally friendly broad-spectrum antimicrobial agent with superior antimicrobial properties [20], including strong antimicrobial activity against both Gram-positive and Gram-negative bacteria, and the ability to bind to negatively charged bacterial cell membranes, resulting in cytoplasmic leakage and thus killing bacteria [21,22]. PHMB has the advantages of high antibacterial activity, low cytotoxicity, and high biocompatibility [23,24], and it has been used as a wound disinfectant and as a foam dressing for the treatment of wound infections [25,26].

Our previous study showed that SF scaffolds can effectively load PHMB, and PHMB can be slowly released in SF scaffolds for up to 20 days, eliciting a certain antibacterial effect [27]. The positively charged PHMB is loaded into the SF scaffold through electrostatic interactions with the negatively charged SF, avoiding the use of chemical crosslinkers, which is beneficial for the cytocompatibility of the PHMB/SF scaffold. However, the loading of high doses of antibacterial agents may have a negative effect on the cytocompatibility of SF scaffolds. Therefore, investigating the effect of the PHMB loading ratio in SF scaffolds on their cytocompatibility is crucial for the clinical application of PHMB/SF scaffolds as a novel antibacterial wound dressing.

The aim of this study was to investigate the extent and correlation of the effects of PHMB loading on the antimicrobial performance and cytocompatibility of PHMB/SF hybrid scaffolds and to determine the threshold PHMB/SF ratio that does not cause a significant negative effect on cell growth in the PHMB/SF hybrid scaffold. PHMB/SF hybrid scaffolds with different PHMB proportions were prepared via freeze-drying, and PHMB was loaded into the scaffold through electrostatic interactions with SF. The relationship between the PHMB/SF ratio and the antibacterial activity of the scaffold against *E. coli* and *S. aureus* was investigated using the agar disc diffusion method and bacteriostasis rate determination. The effect of the PHMB/SF ratio on the growth of L-929 mouse fibroblasts inoculated in the scaffolds was observed via SEM and laser confocal microscopy, and the effect on cell proliferation was examined using a CCK-8 assay.

## 2. Materials and Methods

### 2.1. Preparation of SF Solution

Silk fibers (Nantong, China) were degummed three times in 0.05 wt% Na_2_CO_3_ (Sinopharm Chemical Reagent Co., Ltd., Shanghai, China) aqueous solution at 100 °C for 30 min and dried at 60 °C after being rinsed thoroughly with deionized water. The extracted fibers were dissolved in 9.3 mol/L LiBr (Sigma-Aldrich, Saint Louis, MO, USA) solution at 60 °C for 1 h. Regenerated SF solution was obtained after dialysis (MWCO, 8–14 kDa) in deionized water for 3 days.

### 2.2. Preparation of the PHMB/SF Scaffold

**Figure 1:**
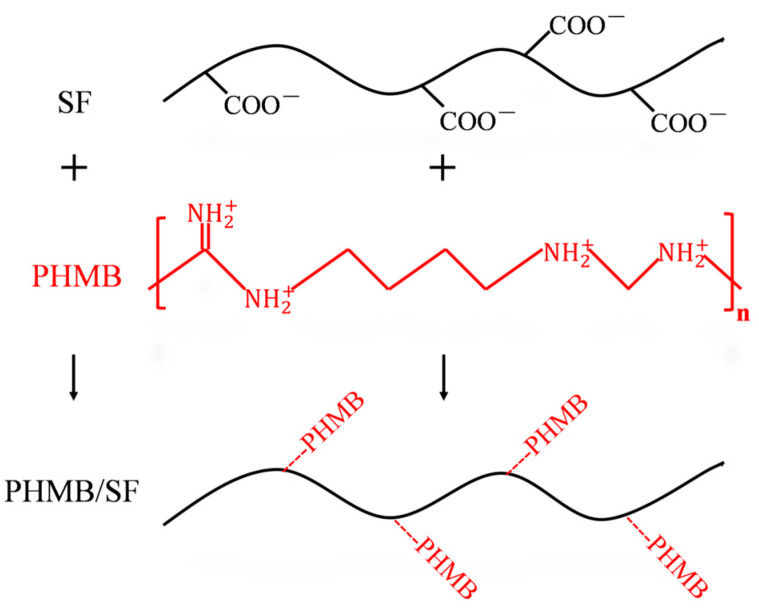
Schematic illustration of the formation of the PHMB/SF complex.

As shown in Figure 1, when positively charged PHMB was blended with the negatively charged SF solution, PHMB bound to SF through electrostatic interactions to form a PHMB/SF complex. Next, PHMB/SF scaffolds were prepared via freeze-drying. The specific method is described below.

An SF solution of 2 wt% was weighed in a beaker, and 10% (*w*/*w*) propanetriol solution was added so that the mass of propanetriol in the mixed solution accounted for 30% of the mass of SF. Then, the aqueous solution of PHMB (Lonza Co., Ltd., Allendale, NJ, USA) was drop-added to the slowly stirred SF solution. The mass ratios of PHMB/SF in the mixed solution were 0/100, 0.5/100, 1/100, 2/100, 3/100, 7/100, and 10/100. The blend solution was cast onto a stainless-steel plate in a layer with a thickness of approximately 2 mm. The stainless-steel plates containing the blend solution were immediately frozen at −40 °C for 6 h and then freeze-dried in a lyophilizer (VirTis, Columbus, OH, USA) for 36 h to prepare PHMB/SF scaffolds. The prepared scaffolds were placed in a constant-temperature and humidity chamber at 40 °C and RH 90% for 24 h to render the scaffold insoluble in water. Finally, the hybrid scaffolds were sterilized via γ-ray irradiation and stored at 2–8 °C.

### 2.3. Morphological Observation of the Scaffolds

PHMB/SF scaffold samples were attached to an electron microscope stage with conductive adhesive and sprayed with gold for 90 s, and the cross-sectional morphology of the hybrid scaffold was observed using scanning electron microscopy (S-4800, Hitachi, Tokyo, Japan).

### 2.4. X-ray Diffraction and Fourier Transform Infrared Spectroscopy of the Scaffolds

X-ray diffraction (XRD) was performed using an X’ Pert-Pro MPD diffractometer (Philips, Almelo, The Netherlands), and CuKα radiation with a wavelength of 1.5406 Å was used. The scanning speed was 2°/mm. The diffraction intensity curves with 2*θ* from 5° to 50° were obtained. The PHMB/SF scaffold was cut into tiny particles and sieved through a 150-mesh screen, and the particles were co-mixed and ground with KBr crystals and pressed into tablets. Fourier transform infrared (FTIR) spectra were obtained using a Nicolet 5700 (Thermo Fisher Scientific Inc., Waltham, MA, USA) in the range of 500–3500 cm^−1^.

### 2.5. Zeta Potential of the PHMB/SF Complexes

PHMB/SF complexes with PHMB/SF mass ratios of 0/100, 0.5/100, 1/100, 2/100, 3/100, 7/100, and 10/100 were prepared via adding PHMB solution dropwise to SF solution. The solutions were diluted with distilled water to 0.1 wt% for zeta potential measurement. The zeta potential of the PHMB/SF complexes was measured at 25 °C using a Zetasizer Nano ZS90 (Malvern Panalytical, Malvern, UK).

### 2.6. Antibacterial Activity Test

The bacteriostasis circle of the samples against *E. coli* and *S. aureus* was measured using the agar disc diffusion method [28]. The PHMB/SF scaffold was cut into uniformly sized discs of 10 mm in diameter, and the SF scaffold was used as a control. All samples were sterilized using ^60^Co irradiation.

*E. coli* and *S. aureus* were grown using Luria−Bertani medium and incubated for 16 h at 37 °C in a shaking incubator (160 rpm/min). The concentration of the bacterial solution was 2 × 10^9^ CFU/mL. The moist scaffolds were placed on the surfaces of agar plates inoculated with bacteria (*E. coli* or *S. aureus*) and incubated for 16 h at 37 °C in an incubator. Finally, the bacteriostasis circle width was calculated using Formula (1).

(1) $$H=\frac{D\;-\;d}{2}$$

Here, H (mm) is the width of the bacteriostasis circle, d (mm) is the sample diameter, and D (mm) is the outer diameter of the bacteriostasis circle.

The bacteriostasis rate (%) of the scaffolds was assessed using the shake flask method [29]. Samples were immersed in 75 mL of PBS solution for three days, and then 5 mL (2 × 10^7^ CFU/mL) of bacterial suspension was added and incubated for 18 h at 37 °C in a shaking incubator (160 rpm/min). The bacterial suspension was then diluted 100-fold, inoculated on agar culture plates, and incubated for 24 h at 37 °C in a biochemical incubator. Images of colony growth were taken at the end of the culture. The number of colonies in the dish was recorded, and the bacteriostasis rate (%) was calculated using Formula (2).

(2) $$X=\frac{W\;-\;Q}{W}\;\times \;100\%$$

Here, X (%) is the bacteriostasis rate, W (CFU) is the average number of colonies in the SF scaffold petri dish, and Q (CFU) is the average number of colonies on the hybrid scaffold petri dish with different PHMB/SF mass ratios.

### 2.7. SEM Observation of Cell morphology

L929 cells (ATCC, Manassas, VA, USA) were cultured using Dulbecco’s modified Eagle medium (Gibco, Waltham, MA, USA) containing 10% fetal bovine serum (Gibco, Waltham, MA, USA). Cells were grown in cell culture dishes using a humidified 5% CO_2_ incubator at 37 °C.

L929 cells were cultured in PHMB/SF hybrid scaffolds for 1, 3, and 7 days, and the medium was discarded. After gently washing the samples with PBS three times, 2.5% glutaraldehyde solution was added and fixed in a 2–8 °C refrigerator for 12 h. Next, dehydration was performed with 50%, 60%, 70%, 80%, 90%, and 100% ethanol in sequence. The dehydrated porous scaffolds were vacuum-dried and sprayed with gold for 90 s, followed by examination using scanning electron microscopy (SEM).

### 2.8. Laser Confocal Microscopy Observation of Cell Morphology

L929 cells were cultured in PHMB/SF hybrid scaffolds for 1, 3, and 7 days, and the medium was discarded. After gently washing the samples with PBS three times, 1 mL of fluorescein diacetate (Sigma-Aldrich, Saint Louis, MO, USA) solution (10 g/mL) was added to each sample, and the samples were incubated in the dark for 10 min. Next, the reaction solution was discarded, and the samples were rinsed thoroughly with PBS. The cell morphology inside the hybrid scaffolds was observed using a laser confocal microscope.

### 2.9. Cell Proliferation Assay

Cell viability evaluations were performed using the CCK-8 assay. L-929 cells were cultured in PHMB/SF scaffolds for 1, 3, and 7 d, and the medium was discarded. After gently rinsing the samples with PBS solution, 500 μL of medium with 50 μL of CCK-8 reagent (Sigma-Aldrich, Saint Louis, MO, USA) was added to each well. The 24-well plates were cultured in an incubator for 3 h. The medium was aspirated into 1.5 mL centrifuge tubes and centrifuged at 1000 rpm for 5 min. The supernatant was added to 96-well plates at 100 μL per well. The optical density (OD) was measured at 450 nm using a microplate reader (Synergy HT, Bio Tek, Winooski, VT, USA). The cell viability of L-929 cells was calculated using Formula (3). Each experiment was performed in triplicate and repeated at least three times.

(3) $$\mathrm{Cell}\;\mathrm{viability}\;\left(\%\right)=\frac{{\mathrm{OD}}_{450(\mathrm{sample})}}{\;{\;\mathrm{OD}}_{450(\mathrm{control})}}\;\times \;100\%$$

Here, OD_450(sample)_ is the optical density of PHMB/SF scaffolds with different mass ratios, and OD_450(control)_ is the optical density of the SF scaffold group.

### 2.10. Statistical Analysis

Data are expressed as the mean ± standard deviation (SD). Samples were evaluated using one-way analysis of variance (ANOVA). Statistical significance was set in advance to a probability level of 0.05.

## 3. Results

### 3.1. Characterization of the PHMB/SF Scaffold

**Figure 2:**
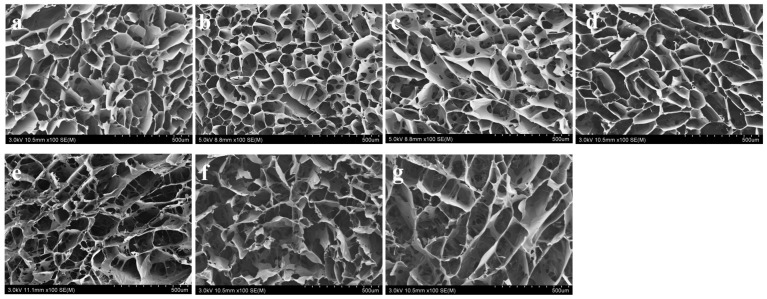
SEM images of the PHMB/SF scaffolds. PHMB/SF mass ratios: (**a**) 0/100; (**b**) 0.5/100; (**c**) 1/100; (**d**) 2/100; (**e**) 3/100; (**f**) 7/100; (**g**) 10/100. Scale bar: 500 μm.

Figure 2 shows cross-sectional SEM images of PHMB/SF hybrid scaffolds with different PHMB/SF mass ratios. The interior of the PHMB/SF scaffolds presented a porous structure. The shape of the pores inside the scaffolds was oval or polygonal, and the average size of the pores was 136~171 μm. No obvious PHMB accumulation was observed on the pore walls. Compared with the SF porous scaffold without PHMB, the shape of the pores in the scaffold did not change significantly after PHMB was incorporated into the SF scaffold. When the PHMB/SF mass ratios were 3/100, 7/100, and 10/100, there were more micron-scale secondary pores on the pore walls of the large pores inside the scaffolds, and the connectivity between the large pores was improved. The formation of secondary pores on the walls of the large pores in the scaffold provided channels for the release of PHMB loaded in the scaffold.

**Figure 3:**
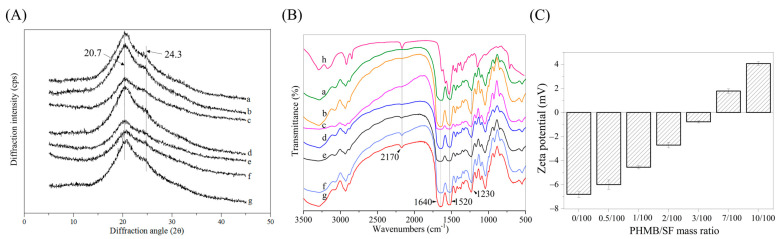
Characterization of the PHMB/SF scaffolds. (**A**) XRD curves. (**B**) FTIR spectra. (**C**) Zeta potential. PHMB/SF mass ratios: (a) 0/100; (b) 0.5/100; (c) 1/100; (d) 2/100; (e) 3/100; (f) 7/100; (g) 10/100; (h) 100/0.

In the X-ray diffraction pattern of the SF scaffold, the diffractions of Silk I appeared at approximately 12.2°, 19.7°, 24.3°, and 28.2°, and the diffractions of Silk II appeared at approximately 9.1°, 18.9°, and 20.7° [30]. Figure 3A shows the X-ray diffraction curves of PHMB/SF scaffolds with different mass ratios. The SF scaffold showed a strong diffraction peak near 20.7° and a medium diffraction peak near 24.3°. These results indicate that the molecular conformation of SF in the scaffold was mainly β-folded after induction treatment with glycerol. Compared with the SF scaffold, the diffraction peak of the PHMB/SF hybrid scaffold did not change significantly in the X-ray diffraction curve, indicating that the addition of PHMB did not significantly affect the molecular conformation of SF.

The FTIR spectra results also showed that PHMB did not have a significant effect on the structure of the scaffold (Figure 3B). The characteristic absorptions of SF at amides I, II, and III in the SF scaffolds appeared at 1640 cm^−1^, 1520 cm^−1^, and 1230 cm^−1^, respectively. Compared with the SF scaffolds without PHMB, the characteristic absorptions of the PHMB/SF scaffold at amides I, II, and III did not change significantly, showing that the molecular conformation of the SF in the hybrid scaffold was dominated by a β-sheet structure [31,32,33].

After the positively charged PHMB was blended with the negatively charged SF, the PHMB bound to SF through electrostatic interactions, forming PHMB/SF complexes. As shown in Figure 3C, the zeta potential of SF was −6.81 ± 0.26 mV, and the zeta potential of the PHMB/SF complex gradually increased with an increasing PHMB/SF mass ratio. When the PHMB/SF mass ratio was 3/100, the zeta potential of the complex was −0.78 ± 0.09 mV, and the negative charge on the SF surface was basically neutralized. When the PHMB/SF mass ratio was 7/100, the surface charge of the complex turned from negative to positive, and the zeta potential of the complex was +1.78 ± 0.19 mV. When the PHMB/SF mass ratio was 10/100, the zeta potential of the complex increased to +4.07 ± 0.15 mV, and the surface of the complex carried a large positive charge. The cumulative release rate of PHMB from the PHMB/SF scaffolds gradually increased with an increasing zeta potential (Figure S1). Although the cumulative release rates of the scaffolds with different mass ratios were different, all the scaffolds showed an initial burst release of PHMB, followed by sustained release. This could facilitate the antibacterial effect of PHMB in the PHMB/SF scaffold for long durations.

### 3.2. Antimicrobial Activity of PHMB/SF Scaffolds

**Figure 4:**
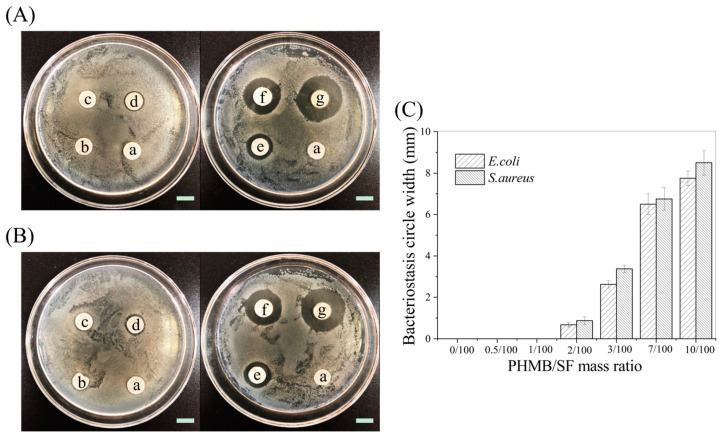
Bacteriostasis circles of hybrid scaffolds with different PHMB/SF mass ratios. (**A**) Images against *E. coli*. (**B**) Images against *S. aureus*. (**C**) Widths of the bacteriostasis circle. PHMB/SF mass ratios: (a) 0/100; (b) 0.5/100; (c) 1/100; (d) 2/100; (e) 3/100; (f) 7/100; (g) 10/100. Scale bar: 10 mm.

Figure 4A,B show images of the bacteriostasis circles of the hybrid scaffolds with different PHMB/SF mass ratios against *E. coli* and *S. aureus*. The SF scaffold without PHMB did not produce bacteriostasis circles, indicating that the SF scaffold without PHMB did not have an inhibitory effect on *E. coli* and *S. aureus*. No significant bacteriostasis circles were observed around the hybrid scaffolds with PHMB/SF mass ratios of 0.5/100 and 1/100. There was an obvious inhibition zone around the hybrid scaffold with a PHMB/SF mass ratio of 2/100, and the widths of the bacteriostasis circles against *E. coli* and *S. aureus* were 0.68 ± 0.11 mm and 0.88 ± 0.18 mm, respectively. When the PHMB/SF mass ratio was 3/100, the widths of the bacteriostasis circles against *E. coli* and *S. aureus* were 2.63 ± 0.18 mm and 3.38 ± 0.18 mm, respectively. When the PHMB/SF mass ratio increased to 10/100, the widths of the PHMB/SF scaffold against *E. coli* and *S. aureus* was further increased to 7.75 ± 0.35 mm and 8.51 ± 0.61 mm, respectively. As shown in Figure 4C, with an increasing PHMB/SF mass ratio, the width of the bacteriostasis circles significantly increased, and the bacteriostatic effect of the hybrid scaffolds was enhanced.

**Figure 5:**
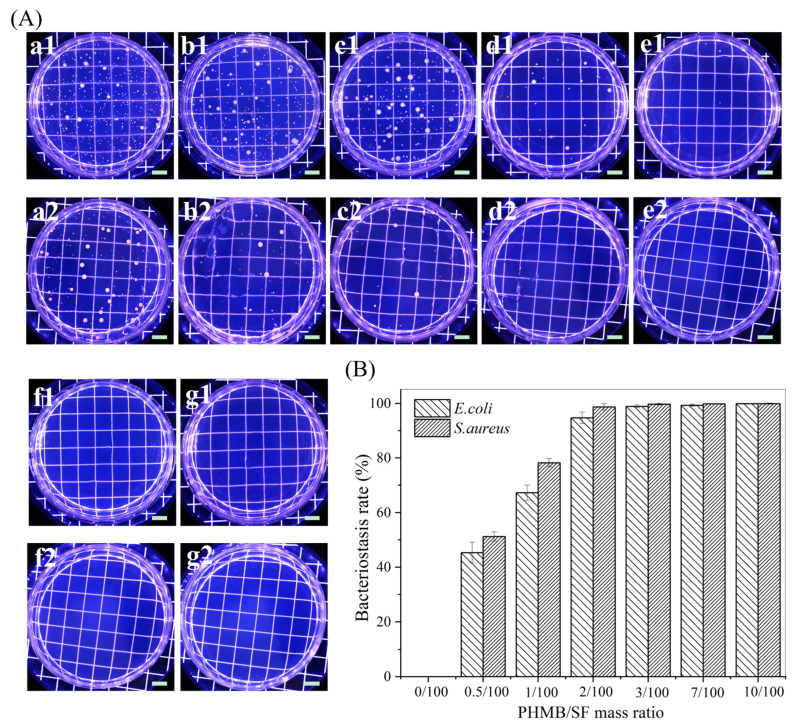
Bacteriostasis rates of hybrid scaffolds with different PHMB/SF mass ratios. (**A**) Images of the bacteriostasis rate test against (**a1**–**g1**) *E. coli* and (**a2**–**g2**) *S. aureus*. (**B**) Bacteriostasis rate. PHMB/SF mass ratios: (**a1**,**a2**) 0/100; (**b1**,**b2**) 0.5/100; (**c1**,**c2**) 1/100; (**d1**,**d2**) 2/100; (**e1**,**e2**) 3/100; (**f1**,**f2**) 7/100; (**g1**,**g2**) 10/100. Scale bar: 10 mm.

The bacteriostasis rates of the PHMB/SF scaffold against *E. coli* and *S. aureus* are shown in Figure 5A. A large number of colonies were found in the Petri dishes of the SF scaffold without PHMB (Figure 5A), and the bacteriostasis rate against *E. coli* and *S. aureus* was 0%. As the PHMB/SF mass ratio increased, the number of colonies gradually decreased. Hybrid scaffolds with PHMB/SF mass ratios of 0.5/100 and 1/100 had bacteriostasis rates against *E. coli* of 45.3 ± 3.8% and 67.2 ± 2.8%, respectively, and those against *S. aureus* were 51.2 ± 1.7% and 78.2 ± 1.5%, respectively (Figure 5B). When the mass ratio of PHMB/SF was 2/100, the bacteriostasis rates of *E. coli* and *S. aureus* were 94.7 ± 2.1% and 98.7 ± 1.2%, respectively. When the PHMB/SF mass ratio was greater than 2/100, few colonies were present in the plates. The bacteriostasis rates of the PHMB/SF scaffolds with mass ratios of 3/100, 7/100, and 10/100 against *E. coli* and *S. aureus* were all close to 100% (Figure 5B). The above results show that the antimicrobial activity of the hybrid scaffolds increased with the increase in the PHMB/SF mass ratio.

### 3.3. Morphology of L-929 Cells in the Scaffold

**Figure 6:**
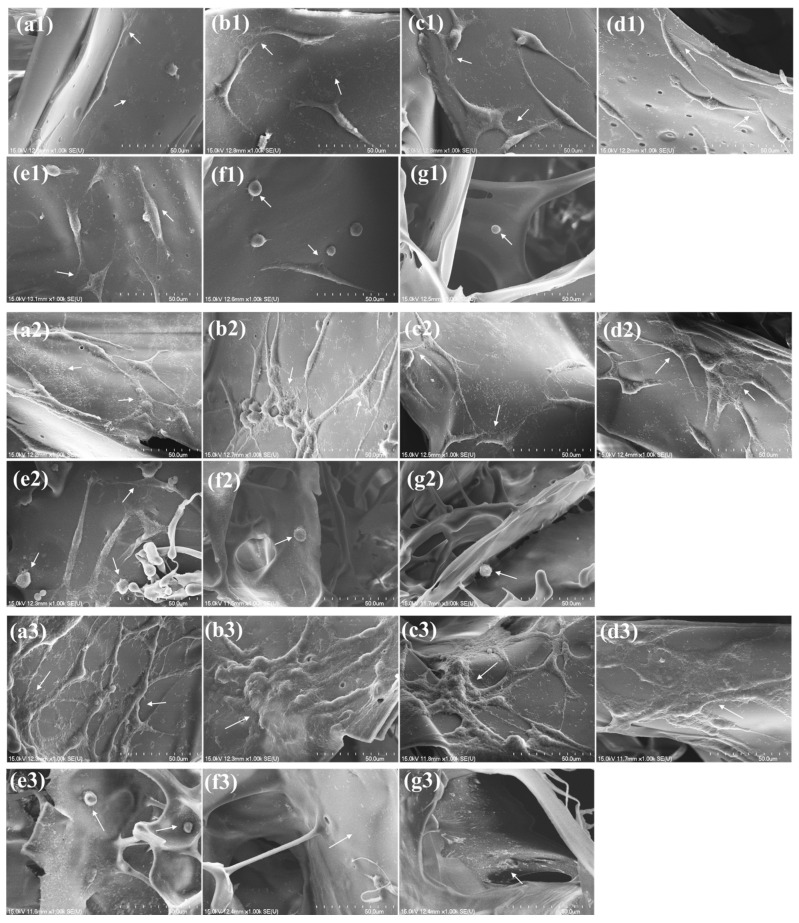
SEM images of L-929 cells seeded in PHMB/SF scaffolds and cultured for 1, 3, and 7 days. (**a1**–**g1**) 1 day; (**a2**–**g2**) 3 days; (**a3**–**g3**) 7 days. PHMB/SF mass ratios: (**a1**–**a3**) 0/100; (**b1**–**b3**) 0.5/100; (**c1**–**c3**) 1/100; (**d1**–**d3**) 2/100; (**e1**–**e3**) 3/100; (**f1**–**f3**) 7/100; (**g1**–**g3**) 10/100. White arrows point to cells. Scale bar: 50 μm.

The morphology of cell adhesion, spreading, and proliferation inside the PHMB/SF scaffolds was observed using scanning electron microscopy (Figure 6). One day after the L-929 cells were seeded, the cells in the SF scaffolds without PHMB were spindle-shaped and could adhere to the surfaces of the scaffolds and extend small numbers of filopodia. The cells in the hybrid scaffolds with PHMB/SF mass ratios of 0.5/100, 1/100, and 2/100 could also adhere to the scaffold surfaces, and their cell morphology was not significantly different from that in the SF scaffold without PHMB. Some of the cells within the hybrid scaffold with a PHMB/SF mass ratio of 3/100 were spherical, and pseudopodia were not apparent. The cells in the PHMB/SF scaffolds with mass ratios of 7/10 and 10/100 were spherical and could not spread.

Three days after inoculation, the number of cells in the SF scaffold increased, and the cells extended a large number of lamellar pseudopodia and fully spread along the walls of the scaffold. Compared with the SF scaffold without PHMB, the morphologies of the cells in the hybrid scaffolds with PHMB/SF mass ratios of 0.5/100, 1/100, and 2/100 were similar, and the number of cells had significantly increased. In the hybrid scaffold with a PHMB/SF mass ratio of 3/100, many cells were spherical, and only a small number of cells were fusiform and could not spread well. The cells in the PHMB/SF scaffolds with mass ratios of 7/10 and 10/100 were still spherical, and the number of cells was small.

At 7 days after inoculation, the number of cells in the SF scaffold without PHMB further increased, and the cells not only fully spread but also connected to each other. Compared with the SF scaffold without PHMB, the number of cells in the hybrid scaffolds with PHMB/SF mass ratios of 0.5/100 and 1/100 was greater, the cells were spreading to a greater degree, and the cells were more tightly connected. The number of cells in the 2/100 PHMB/SF scaffold was not significantly different from that in the SF scaffold, but the cells had spread to a greater degree and were more closely connected. The number of cells in the hybrid scaffolds with PHMB/SF mass ratios of 3/100, 7/100, and 10/100 was small, and the cells could not spread normally.

The SEM results showed that compared with the cells in the SF scaffold without PHMB, the cells in the hybrid scaffolds with PHMB/SF mass ratios of 0.5/100, 1/100, and 2/100 had more abundant pseudopods, exhibited more extensive spreading, and had more obvious intercellular connections, suggesting that the hybrid scaffolds with PHMB/SF mass ratios of 0.5/100, 1/100, and 2/100 can promote cell adhesion, spreading, and proliferation. When the PHMB/SF mass ratio was greater than 3/100, the cells could not spread, proliferate, or grow normally in the hybrid scaffolds.

### 3.4. Proliferation of L-929 Cells in the Scaffold

**Figure 7:**
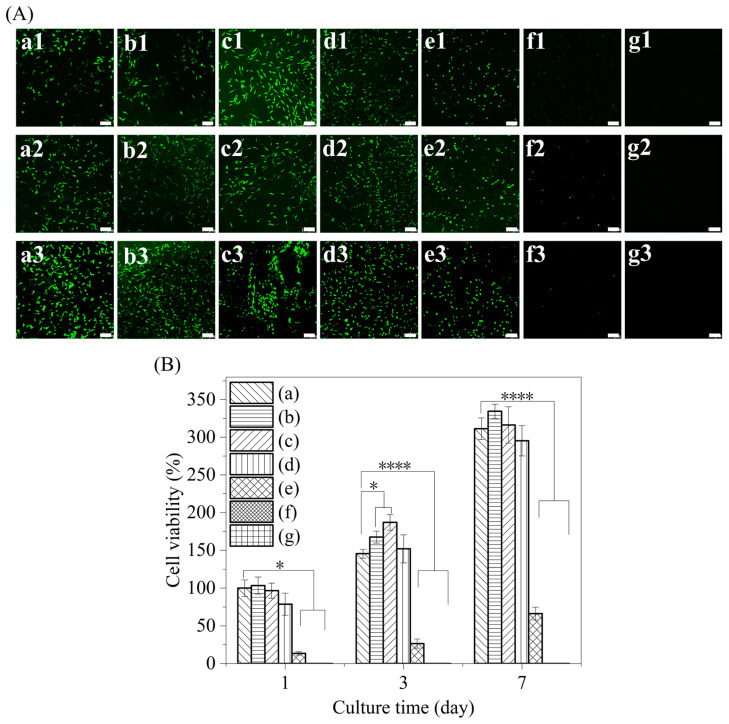
Proliferation of L-929 cells in PHMB/SF scaffolds. (**A**) Laser confocal images of L-929 cells seeded in PHMB/SF scaffolds and cultured for 1, 3, and 7 days. (**a1**–**g1**) 1 day; (**a2**–**g2**) 3 days; (**a3**–**g3**) 7 days. (**B**) Cell viability. PHMB/SF mass ratios: (a) 0/100; (b) 0.5/100; (c) 1/100; (d) 2/100; (e) 3/100; (f) 7/100; (g) 10/100. Scale bar: 100 μm. *: *p* < 0.05, ****: *p* < 0.0001.

The proliferation of L-929 cells cultured in scaffolds with different PHMB/SF mass ratios for 1, 3, and 7 days was observed using laser confocal microscopy (Figure 7A). After 1 day of culturing the L-929 cells in the scaffold, obvious green fluorescence was observed in the SF scaffolds without PHMB and in the PHMB/SF scaffolds with mass ratios of 0.5/100, 1/100, 2/100, and 3/100, indicating the existence of a large number of living cells. Fluorescence was stronger in the PHMB/SF scaffolds with mass ratios of 0.5/100 and 1/100 than in the SF scaffolds without PHMB, showing that more living cells were present in the scaffolds. Weak green fluorescence was observed within the PHMB/SF scaffolds with mass ratios of 7/100 and 10/100, indicating that only a small number of cells had survived.

After 3 days of cell culture, the fluorescence within the PHMB/SF scaffolds with mass ratios of 0.5/100, 1/100, and 2/100 was stronger than that in the SF scaffold without PHMB (Figure 7A). This showed that PHMB/SF scaffolds with mass ratios of 0.5/100, 1/100, and 2/100 had a greater number of viable cells and faster rates of cell proliferation. When the mass ratio of PHMB/SF was 3/100, obvious fluorescence was also observed in the scaffold, but the fluorescence was weaker than that in the SF scaffold without PHMB. Weak green fluorescence was observed in the PHMB/SF scaffolds with mass ratios of 7/100 and 10/100, showing that the cells could not proliferate efficiently.

After 7 days of cell culture, the fluorescence within the SF scaffold without PHMB and in the PHMB/SF scaffolds with mass ratios of 0.5/100, 1/100, and 2/100 was further enhanced compared with that after 3 days of culture (Figure 7A). The fluorescence intensity of the PHMB/SF scaffold with a mass ratio of 3/100 was weaker than that of the SF scaffold without PHMB. Only weak green fluorescence was observed in the PHMB/SF scaffolds with mass ratios of 7/100 and 10/100, showing that the cells could not proliferate effectively.

The proliferative activity of L-929 cells in the scaffolds determined via the CCK-8 assay is shown in Figure 7B. After 1 day of culture, the cell viabilities of the PHMB/SF scaffolds with mass ratios of 0.5/100, 1/100, 2/100, 3/100, 7/100, and 10/100 were 103.4%, 96.7%, 78.7%, 13.4%, 0%, and 0%, respectively. Compared with the cell viability of the SF scaffold without PHMB (100%, Figure 7B(a)), the cell viability of the PHMB/SF scaffolds with mass ratios of 3/100, 7/100, and 10/100 decreased significantly. After 3 days of culture, the cell viabilities of the PHMB/SF scaffolds with mass ratios of 0.5/100 and 1/100 were 167.4% and 186.8%, respectively, which were significantly higher than the cell viability of the SF scaffold without PHMB (145.3%, *p* < 0.05). The cell viability of the PHMB/SF scaffold with a mass ratio of 2/100 was 151.5%, which was not significantly different from that of the SF scaffold without PHMB. The cell viabilities of the scaffolds with mass ratios of 3/100, 7/100, and 10/100 were 26.4%, 0%, and 0%, respectively, and these values are significantly lower than the cell viability of the SF scaffolds (*p* < 0.0001). After 7 days of culture, the cell viabilities of the PHMB/SF scaffolds with mass ratios of 0.5/100, 1/100, 2/100, 3/100, 7/100, and 10/100 were 334.5%, 316.3%, 295.3%, 66.2%, 0%, and 0%, respectively. Compared with the cell viability of the SF scaffold group (311.3%), the cell viability of the PHMB/SF scaffolds with mass ratios of 3/100, 7/100, and 10/100 was significantly lower, while the cell viability of the PHMB/SF scaffolds with mass ratios of 0.5/100, 1/100, and 2/100 was not significantly different.

The results of the CCK-8 assay were consistent with the results of laser confocal microscopy observation, showing that PHMB/SF scaffolds with mass ratios of 0.5/100 and 1/100 could better promote cell proliferation compared with the SF scaffolds without PHMB. The PHMB/SF scaffold with a mass ratio of 2/100 did not have a significant effect on cell proliferation compared with the SF scaffold without PHMB. When the mass ratio of PHMB/SF was greater than 3/100, however, the proliferation activity of cells in the PHMB/SF scaffold decreased significantly.

## 4. Discussion

Open wounds provide favorable conditions for microbial colonization, and once wounds are infected with bacteria, the normal healing process is seriously compromised [34,35]. Systemic antimicrobial agents do not work well because of poor blood circulation at the site of the wound. It is therefore essential to use a wound covering that prevents bacterial infection [36,37]. In this study, PHMB/SF hybrid scaffolds with an antimicrobial function were prepared via freeze-drying based on electrostatic interactions between PHMB and SF. The experimental results showed that when the PHMB/SF ratio was approximately 2/100, the hybrid scaffold not only had a strong antibacterial effect but also promoted cell spreading and proliferation. When this scaffold is used to cover a wound, depending on the PHMB carried by the hybrid scaffold, it will be able to continuously inhibit bacterial reproduction in the local wound and guide the repair of the wound through the three-dimensional microenvironment inside the scaffold.

The loading of PHMB in SF scaffolds changed the zeta potential of SF but had no significant effect on the structure of the SF scaffolds (Figure 2 and Figure 3). PHMB is positively charged and can bind to negatively charged SF. As the mass ratio of PHMB increased, the zeta potential of SF protein gradually changed from negative to positive (Figure 3C). This showed that PHMB effectively neutralized the negative charges on the surface of the SF via electrostatic interactions.

SF scaffolds have good biocompatibility and can promote cell adhesion and proliferation, and they have been widely used in the field of tissue engineering. However, SF scaffolds do not have antibacterial activity, which limits their applications regarding open wounds. The PHMB/SF scaffolds prepared in this study were able to release PHMB slowly and sustainably (Figure S1) and exhibited effective antimicrobial activity (Figure 4 and Figure 5). As a broad-spectrum antibacterial agent, PHMB itself has a positive charge, and its combination with the negatively charged bacterial cell wall leads to the leakage of bacterial cytoplasm, thereby killing bacteria [38]. The PHMB/SF scaffold’s inhibition of *S. aureus* was stronger than that for *E. coli* (Figure 4 and Figure 5). The cell wall of *S. aureus* is composed of peptidoglycans with abundant pores, making it more susceptible to PHMB attacks, resulting in cell fragmentation. However, the outer cell wall of *E. coli* is composed of lipopolysaccharides, lipoprotein, and phospholipids, which have a certain degree of resistance to the attack of PHMB [39]. The concentration of PHMB solution directly used in clinical wound disinfection is 0.2–1.0 mg/mL [40]. After a 12 h immersion in phosphate-buffered saline (pH7.4), the cumulative release ratio of PHMB in the hybrid scaffold with a PHMB/SF ratio of 10/100 was about 51.9% (Figure S1), resulting in a PHMB concentration of approximately 1.0 mg/mL in the liquid contained in the scaffold, which reached the maximum dose for wound disinfection. Therefore, the maximum mass ratio of PHMB/SF in the hybrid scaffold was set as 10/100 in this study.

The PHMB/SF scaffold has a porous structure with a pore size of approximately 163~171 μm, which provides a three-dimensional space for cell adhesion and proliferation (Figure 2). After neutralizing the partial negative charge on the SF surface with PHMB, the electrostatic repulsion between the surface of the pore wall inside the scaffold and the cell surface was reduced, and cell adhesion and spreading were further promoted (Figure 6). The results of the cell proliferation experiment also showed that the cells on the PHMB/SF scaffolds with mass ratios of 0.5/100, 1/100, and 2/100 had effective proliferation activity (Figure 7). Unlike medical antibiotics, PHMB can directly destroy the bacterial wall and hinder the metabolism of bacteria, rather than interfering with the synthesis of the bacterial wall, which may be an important reason why PHMB does not have obvious cytotoxicity, genotoxicity, and bacterial resistance [41,42].

Antimicrobial agents, such as nano silver particles, antibiotics, antimicrobial peptides, and cationic polymers, are effective in conferring antibacterial properties on dressings (Table 1). Nano silver particles have a strong antibacterial effect, but they also exert cytotoxicity and hepatotoxicity, which have adverse effects on the cytocompatibility of dressings [43,44,45]. Antibiotics and antimicrobial peptides have antibacterial effects [18,46,47,48,49], but there are problems regarding bacterial resistance [50]. Antimicrobial peptides are usually bound to dressings via chemical crosslinking, and the residue of crosslinking agents potentially endows dressings with cytotoxicity [51]. Although cationic compounds such as polyvinylimide and chitosaccharides have antibacterial effects to some extent, they also pose a risk of cytotoxicity [52,53]. In this study, PHMB, with low cost and no risk of long-term toxicity or bacterial resistance, was chosen as an antimicrobial agent. PHMB was loaded into the scaffold through electrostatic interactions without the use of cross-linking agents, and this was beneficial to improving the cytocompatibility of the scaffold. The results of the cytocompatibility experiments showed that the loading of PHMB applied to the scaffolds not only promoted cell proliferation but also facilitated cell adhesion (Figure 6 and Figure 7).

In the PHMB/SF hybrid scaffold constructed in this study, the SF components mainly play three roles. One is to provide adhesion sites for cell growth and promote cell proliferation. Secondly, silk fibroin scaffolds have the ability to contain water, and a moist environment is conducive to skin repair. Finally, as the carrier of PHMB, a fibrin scaffold can avoid an excessive explosive release of PHMB and achieve continuous release of PHMB. Our previous studies have shown that 1, 2, 3, and 4 weeks after the implantation of freeze-dried SF scaffolds into the full-thickness skin defect wounds of SD rats, the degradation rates were 28%, 59%, 74%, and 85%, respectively, which basically matched the speed of wound repair [54]. Therefore, when the PHMB/SF scaffold covers a wound, the potential functions of sustained antibacterial activity and the promotion of wound repair can be expected.

Compared with the PHMB-free SF scaffolds, cell viability was significantly increased in the hybrid scaffolds with PHMB/SF mass ratios of 0.5/100 and 1/100, while cell viability was significantly decreased in the hybrid scaffolds with PHMB/SF mass ratios of 3/100, 7/100, and 10/100 (Figure 7). PHMB can induce cell membrane damage at high concentrations, change the permeability of the cell membrane, and induce cell apoptosis [41,55]. Low concentrations of PHMB exhibit no obvious cytotoxicity [56], and PHMB can shield part of the negative charge on the SF surface, reduce the repulsion of SF on the negatively charged cell surface, and enhance cell adhesion, spreading, and proliferation, thereby creating a microenvironment for wound healing.

## 5. Conclusions

The results of antibacterial activity testing showed that the hybrid scaffolds with PHMB/SF mass ratios of 2/100, 3/100, 7/100, and 10/100 had effective antibacterial functions. The results of the in vitro culture of L-929 cells seeded into scaffolds showed that the hybrid scaffolds with PHMB/SF mass ratios of 0.5/100 and 1/100 not only lacked a negative effect on cell growth but also unexpectedly promoted cell adhesion, spreading, and proliferation. Compared with the SF scaffold without PHMB, the hybrid scaffolds with a PHMB/SF mass ratio of 2/100 had no significant negative effects on the spreading and proliferation of seeded cells. In conclusion, when the mass ratio of PHMB/SF is approximately 2/100, the hybrid scaffold has both effective bacteriostatic properties and good cytocompatibility, indicating its potential to be used as a novel antibacterial biocompatible dressing for the treatment of open and infected wounds.

## Figures and Tables

**Table 1:** Antibacterial efficiency and cytocompatibility of SF-based dressings.

Antibacterial Agent	Material Form	Loading Method	Antimicrobial Efficiency	Cytocompatibility	Ref.
Nano silver	Hydrogel	Droplet microfluidic approach	*E. coli* (75%)	Cell viability exceeded 97%	[43,44,45]
	Mat	Coating	Zone of inhibition (12 h): *S. aureus* (10 mm) *E. coli* (14 mm)	Reached to confluence stage after 5 days of incubation	
	Hydrogel	Hydrothermal	Growth inhibition of *E. coli*	Can promote cell proliferation	
Antibiotics	Film	Dipping	Zone of inhibition (24 h): *S. aureus* (19–21 mm) *E. coli* (16–18 mm)	Reached to confluence stage after 5 days of incubation	[18,46,47,48]
	Film	Electrospinning	Zone of inhibition (24 h): *S. aureus* (3 mm)	No cytotoxicity	
	Scaffold	Freeze-drying	Zone of inhibition (26 d): *S. aureus* (11–14 mm)	No harmful effect on cell viability	
	Scaffold	Dipping	Zone of inhibition (24 h): *S. aureus* (8.8 mm) *E. coli* (7.5 mm)	Can promote wound healing	
Antibacterial peptides	Bioadhesives	Blended	Zone of inhibition (12 h): *S. aureus* (2.1 mm) *E. coli* (4.3 mm)	Cell viability approached 100%	[16,17,49,51]
	Scaffold	Electrospinning	Zone of inhibition (12 h): *S. aureus* (7.8 mm) *E. coli* (6.9 mm)	Cell viability approached 90%	
	Film	Covalent cross-linking	*S. aureus* (75%)	Cell viability decreased	
	Film	Covalent cross-linking	Strong antibacterial properties against *S. epidermidis*, *E. coli*	No cytotoxic	
Cationic polymers	Film	Electrospinning	*E. coli* (93.63 ± 2.09%)	No obvious cytotoxicity	[14,15,52,53]
	Film	Electrospinning	Complete inhibition of *S. aureus* and *P. aeruginosa* growth after 5 h	No obvious cytotoxicity	
	Scaffold	Blended	Zone of inhibition (24 h): E. coli (16 mm) *Pseudomonas aureus* (14 mm)	No obvious cytotoxicity	
	Film	Electrostatic interaction	*E. coli* and *S. aureus* (98%)	Cell viability approached 78%	

## Data Availability

The data that support the findings of this study are available from the corresponding author upon reasonable request.

## Supplementary Materials

The following supporting information can be downloaded at: https://www.mdpi.com/article/10.3390/polym15234581/s1, Figure S1: PHMB release within 20 d from hybrid scaffolds.
