# Supplementary figures and images for: Preparation and Properties of Antibacterial Silk Fibroin Scaffolds
Source: Polymers (Basel). 2023 Nov 30;15(23):4581. doi: 10.3390/polym15234581 (PMC10708750; doi:10.3390/polym15234581)

## Supplementary materials

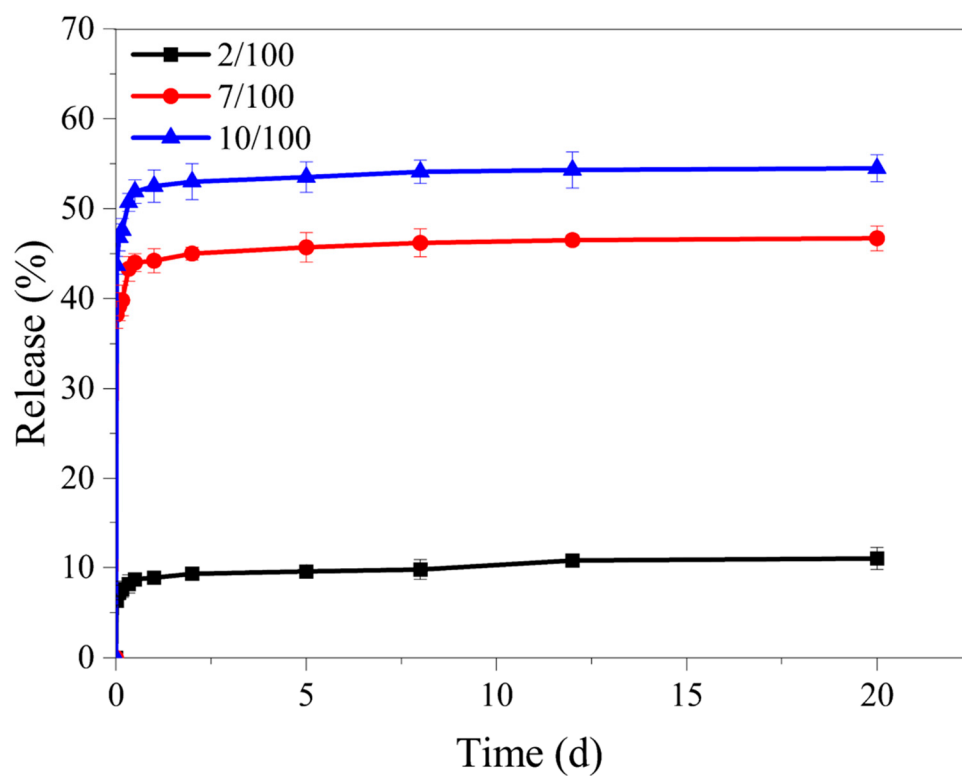

Figure S1. PHMB release within 20 d from hybrid scaffolds.

Supplement: Supplementary file 1 [file polymers-15-04581-s001.zip › polymers-2729279-supplementary.pdf]
